# A PVDF-Based Sensor for Internal Stress Monitoring of a Concrete-Filled Steel Tubular (CFST) Column Subject to Impact Loads

**DOI:** 10.3390/s18061682

**Published:** 2018-05-23

**Authors:** Guofeng Du, Zhao Li, Gangbing Song

**Affiliations:** 1 School of Urban Construction, Yangtze University, Jingzhou 434023, China; gfdu@yangtzeu.edu.cn (G.D.); zhaoliwut@163.com (Z.L.); 2 Department of Mechanical Engineering, University of Houston, Houston, TX 77204, USA

**Keywords:** concrete-filled steel tubular (CFST) column, PVDF (Polyvinylidene Fluoride), piezoelectric sensor, impact sensor, embeddable sensor, impact test, internal stress monitoring

## Abstract

Impact loads can have major adverse effects on the safety of civil engineering structures, such as concrete-filled steel tubular (CFST) columns. The study of mechanical behavior and stress analysis of CFST columns under impact loads is very important to ensure their safety against such loads. At present, the internal stress monitoring of the concrete cores CFST columns under impact loads is still a very challenging subject. In this paper, a PVDF (Polyvinylidene Fluoride) piezoelectric smart sensor was developed and successfully applied to the monitoring of the internal stress of the concrete core of a CFST column under impact loads. The smart sensor consists of a PVDF piezoelectric film sandwiched between two thin steel plates through epoxy. The protection not only prevents the PVDF film from impact damages but also ensures insulation and waterproofing. The smart sensors were embedded into the circular concrete-filled steel tube specimen during concrete pouring. The specimen was tested against impact loads, and testing data were collected. The time history of the stress obtained from the PVDF smart sensor revealed the evolution of core concrete internal stress under impact loads when compared with the impact force–time curve of the hammer. Nonlinear finite element simulations of the impact process were also carried out. The results of FEM simulations had good agreement with the test results. The results showed that the proposed PVDF piezoelectric smart sensors can effectively monitor the internal stress of concrete-filled steel tubular columns under impact loads.

## 1. Introduction

Concrete-filled steel tubes (CFSTs), which combine the superior performances of steel and concrete, have been widely researched and applied in civil engineering [1,2]. The load bearing capacity of a CFST structure for normal static loads is less than its capacity against short duration dynamic loads, such as impact loads [3,4,5,6]. The structural deformation and the evolution of internal stress under an impact load are also different from those under static loads or long duration dynamic loads. Taking CFST piers and CFST piles as examples, these CFST structures are basically subject to axial impact loads during the construction process with the hammering method. It is of great meaning to develop a sensor which can monitor the internal stress of the concrete core to analyze the mechanical behavior and failure mechanism of CFST structures.

At present, common stress monitoring devices include strain gauges, Fiber Bragg Grating (FBG) sensors [7,8], ultrasonic sensors, X-ray, elasto-magneto-electric (EME) sensors [9], and piezoelectric sensors [10,11]. Xu et al. [12] proposed a fiber sensor to monitor the three-dimensional force, force distribution, and relative micro-deformation trends of structures. The research showed that the sensor could measure three-dimensional force accurately and effectively, and it had good linearity, repeatability and consistency. Mckeeman et al. [13] presented Fiber Bragg Grating sensors with a metal package to monitor pre-stress in pre-stressed concrete structures. The results showed that the strain sensors could be used to monitor stress levels in irradiated and harsh industrial environments. Yeager et al. [14] presented a new method to monitor the torque in a bolt by using an embedded FBG sensor. The results showed that the preload-sensitive feature increased monotonically with the applied torque. They also demonstrated that the sensor could be used as a uniaxial strain sensor. In general, FBG sensor systems have relatively low bandwidths and may not be best suited for impact applications. Strain gauges are mature and widely used strain sensors, which measure strain by observing changes in their own resistance. However, a strain gauge requires a signal condition system. There is a type of ultrasonic sensor that is based on the linear relationship between the ultrasonic wave velocity and the applied stress. The residual stress of the structure can be measured by this ultrasonic method [15,16]. However, disadvantages of this method include the requirement of surface finish and the use of a coupling agent to ensure sufficient acoustic coupling. The X-ray method is based on the fact that X-rays can directly measure the interatomic spacing, based on which the elastic strain of the metal crystal can be obtained, and the total stress can be measured [17]. However, the disadvantages of this method include radiation, bulky equipment, and unsuitability for real time monitoring.

Piezoceramic materials, such as PZT (Lead Zirconate Titanate), have strong piezoelectric effects [18] and wide bandwidth and are widely used in research and practice as sensors in dynamic applications [19,20,21,22]. Song et al. encapsulated a PZT patch in a concrete block and developed the smart aggregate (SA) [23,24,25]. SAs embedded into a concrete bridge have the potential to not only monitor the impact of over-height vehicles on the bridge, but also evaluate the damage to the structure. The results from SA testing have shown that the output voltage of SA increases linearly with an increase in the impact force. Xu et al. proposed an experimental calibration method to measure the sensitivity of a shear type PZT sensor [26]. The results showed that the shear type PZT smart aggregate responded linearly with the impact loads and different smart aggregates have similar sensitivity characteristics. Hou et al. developed the piezoelectric smart aggregate based on PZT to monitor seismic compressive stress and shear stress [27,28]. The test results showed that the sensitivity of this smart aggregate is constant in the range of the shear strength of concrete, and the method of shear stress monitoring was demonstrated to be feasible. In addition, PZTs are often used in energy harvesting (EH) [29,30,31,32] and structural health monitoring (SHM) [33,34,35,36,37], as well as integration of the two: EH-SHM [38,39]. Furthermore, when a piezoceramic transducer is bonded on, or embedded in, a structure, its electric impedance is coupled with the mechanical impedance of the structure, based on which the conditions of the structure can be monitored [40,41]. However, PZTs, in general, have some disadvantages, such as brittleness and poor impact resistance. For impact sensing applications, the PZTs’ advantage of having a strong piezoelectric effect turns into a disadvantage since PZTs often generate high voltages that exceed the limits of data acquisition systems due to their high piezoelectric coefficient. Therefore, during an impact event, a PZT sensor often suffers from saturation.

PVDF (Polyvinylidene Fluoride), a type of piezoelectric polymer, overcomes the difficulties of PZTs and also has the advantages of having a flexible structure and stable performance [42,43,44], even for energy harvesting [45,46]. For impact applications, the disadvantage in PVDF of having low piezoelectric coefficient actually turns into an advantage in the sense that the sensor does not suffer from voltage saturation when measuring an impact load. In addition, PVDF films are very low cost, compared with PZTs. PVDF films have found many applications [47,48,49,50,51], including structural health monitoring [52]. Shirinov et al. developed a pressure sensor with PVDF film as the sensing element and studied the sensing performance of the sensor [53]. The results showed that the output voltage of the PVDF sensor is several volts, and this can be acquired by common voltage acquisition instruments. Yu et al. studied the sensitivity and frequency response characteristics of a PVDF sensor to monitor natural frequencies of, and damage to, a bridge [54]. The test results showed that PVDF piezoelectric films have a high impact resistance and a fast, dynamic response. Meng and Yi conducted axial impact experiments on concrete columns with an embedded PVDF stress sensor by using a drop hammer [55]. The experimental results demonstrated that the PVDF sensor has a good reliability as a stress sensor. Jeon et al. bonded PVDF on the surface of an aluminum alloy beam that was excited under a sweep sine signal from 0 Hz to 5 kHz to obtain the frequency responses of the beam [56]. The experimental results showed that PVDF performs well in the measurement of dynamic responses and the monitoring of structural vibration. Dung and Sadaki carried out an experimental study and numerical simulation of cantilever beams attached with PVDF sensors under impact loadings [57]. The test results showed that the PVDF sensor captured the fundamental frequencies of the cantilever beam. Huang and Ma carried out a free fall impact test and numerical simulation of cantilever beams attached with PVDF sensors [58]. The results showed that the PVDF film sensor has the ability to capture impact loading processes. In general, like any piezoelectric sensor, a PVDF sensor is mainly used for dynamic applications. Due to the capacitance nature of a piezoelectric sensor, the generated charge will discharge during a static test, and therefore, a charge amplifier is needed if a PVDF is used in static tests.

The above research indicates that PVDF films perform well as sensing elements for impact applications, potentially including the internal stress monitoring of CFSTs under impact loads. However, to the best knowledge of the authors, there has been no previously reported research regarding the use of PVDF film sensors in such applications, which motivated us to conduct research involving the PVDF film sensors to monitor the internal stress of CFSTs during impact. To achieve this goal, a PVDF film sensor had to be designed so that it can be easily deployed as an embedded sensor with proper insulation and energy absorption to survive impact events. This paper describes the design of a PVDF impact sensor to meet these requirements. The core of the sensor is a PVDF film coated with a PET (Polyethylene Terephthalate) plastic layer to provide waterproofing, electric insulation, and impact energy absorption. The core is then sandwiched between two thin steel plates to achieve high strength, strong toughness and easy deployment. Impact experiments of CFST specimens with the embedded PVDF sensors were conducted. In addition, finite element simulations were also performed. Based on the experiments and finite element simulation, it was found that the time history curves of the impact stress were in good agreement with the simulated ones. 

## 2. Experimental Setup

### 2.1. The PVDF Smart Sensor

**Figure 1:**
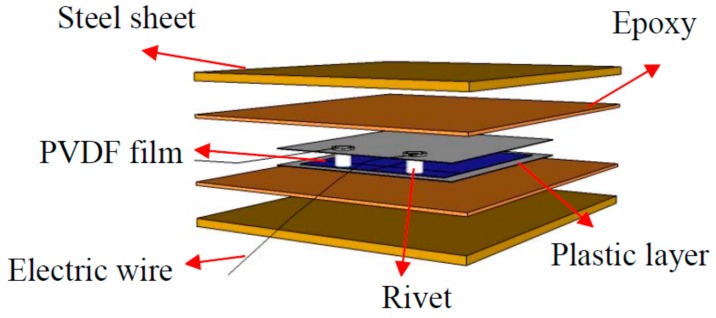
Structure diagram of the Polyvinylidene Fluoride (PVDF) smart sensor.

**Figure 2:**
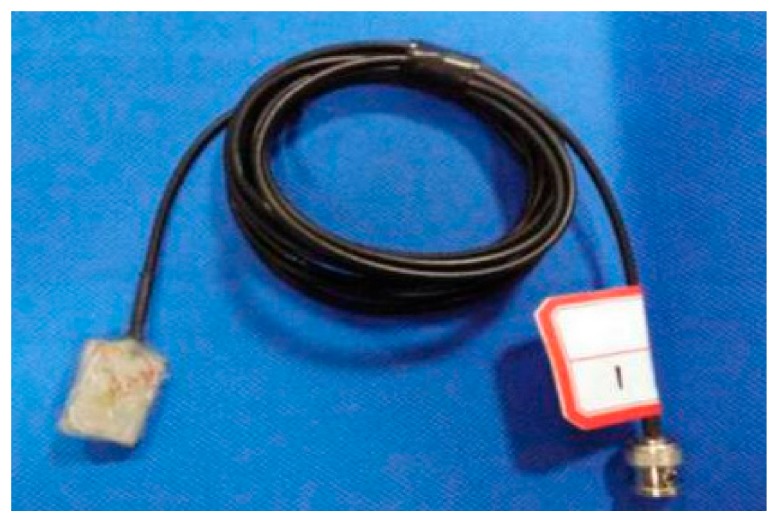
The PVDF smart sensor.

Figure 1 shows the structure of the proposed PVDF smart sensor. The developed sensor uses a PVDF film as a sensing element which is insulated and protected by plastic layers. The plastic layer is a PET film with good toughness, tensile strength, and impact resistance, which is important for absorption of the shock energy to the sensor during impact events. The size of the PVDF film is 10.0 mm × 10.0 mm × 0.03 mm. The electric wires are connected though two flat rivets fixed on the PVDF film to ensure good contact between the electric wires and the film. Then, the smart senor is sandwiched between two steel sheets by epoxy. The steel sheet was chosen as the packaging material, and the size is 22 mm × 18 mm × 0.3 mm. The fabricated sensor is shown in Figure 2. Through this design, the packaged PVDF sensor has high durability to allow it to work effectively under impact loads, and the sensor can be embedded in a concrete structure to monitor internal impact stress. The packaged sensor has toughness and elasticity that is able to handle a stress level of up to 80 MPa to prevent damage to the PVDF film during impact events. In addition, the sensor design is very simple and low cost (the direct cost is about a few US dollars each).

### 2.2. Experimental Setup

Circular CFST specimens with diameters of 140 mm and heights of 360 mm were used for the impact tests. The wall thicknesses of the steel tubes were 2.0, 3.0 and 4.0 mm, respectively. The sensitivity of each sensor was calibrated before embedding. The sensitivity of each sensor is presented in Table 1.

**Figure 3:**
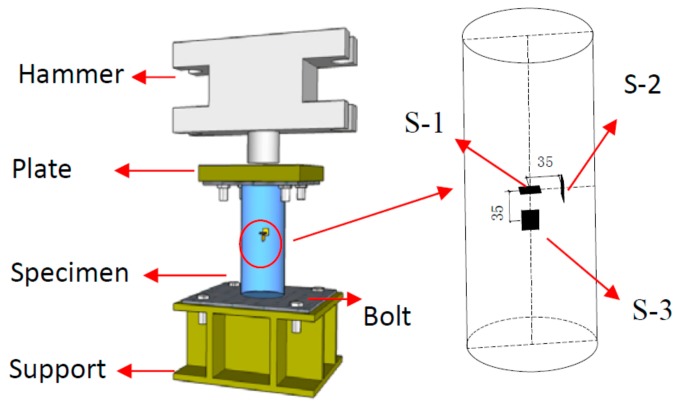
Location of the PVDF smart sensors.

**Figure 4:**
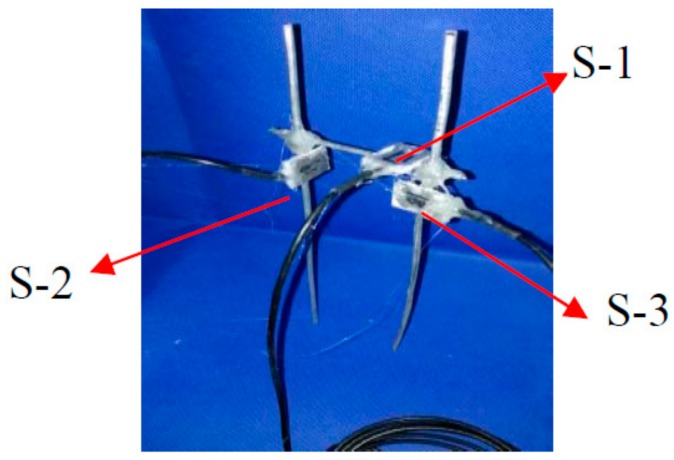
The configuration of sensors prior to embedment.

**Figure 5:**
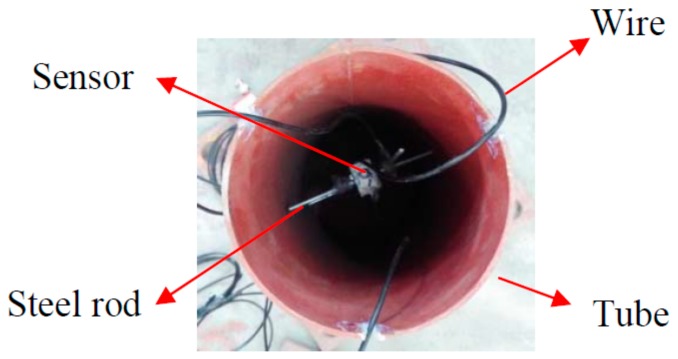
Sensors in a steel tube prior to concrete casting.

**Figure 6:**
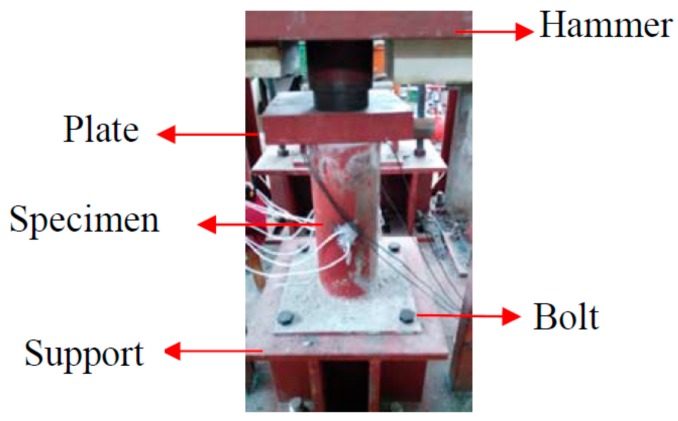
Experimental setup.

There were three PVDF smart sensors embedded in each CFST specimen (as shown in Figure 3) to measure the internal stresses along the three orthogonal directions. Please note that sensor S-1 was placed in the center of the specimen and was used to monitor the vertical internal stress. Sensors S-2 and S-3 were used to monitor the confining stress in the two lateral directions. Figure 4 shows the configuration of the three sensors on a support. The support helped to maintain the sensors’ positions and orientations during concrete pouring and vibration. Figure 5 shows the three sensors in the steel tube prior to the pouring of concrete. After pouring the concrete, the CFST specimens were cured at room temperature for 28 days. Figure 6 is a photo of a fabricated CFST specimen with embedded PVDF sensors.

Compressive strength tests of the three cubes, which were cast from the same concrete and cured under the same conditions, were conducted. The average compressive strength was 25.2 MPa. The yield strength and modulus of elasticity of the steel were also measured. Based on the performance test of the steel, the yield strength, ultimate strength and elastic modulus were 261 MPa, 393 MPa and 182 GPa.

### 2.3. Experimental Procedures

The tests involving impact loads of different heights were carried out using a drop hammer impact test machine. The maximum effective impact height of the drop weight test machine was 7.5 m. The weight of the hammer could vary from 339 to 639 kg through the addition of removable 50 kg loading plates. The impact tests involved 7 working conditions based on the height of the hammer, from 1.0 to 4.0 m. The preload test was necessary to ensure that the impact energy could be well transmitted to the entire specimen, due to the fact that part of impact energy was dissipated in the poor contact area where a contact gap between the top surface of the concrete and the cover plate existed. There were imperfect contacts between the top plate and the top surface of the concrete core of the specimen, i.e., there were small voids and gaps between the top plate and the top surface of the concrete core of the specimen. For the first impact (the impact height was 0.5 m), part of the impact energy was dissipated in the poor contact area and the remaining energy was transferred to the CFST column specimen in the impact process, which resulted in much less impact stress, as reported by the PVDF sensors. Therefore, the impact when *h* = 0.5 m was regarded as the preload test and was used to ensure good contact between the top plate and the top surface of the concrete core of the specimen.

The main monitoring parameters included the output voltages of the accelerometer on the hammer and the PVDF smart sensors under different loading conditions. The acceleration of the hammer in the impact process was obtained using the output voltage of the accelerometer that was fixed on the hammer. The impact force of the hammer in the process of impact was calculated by the multiplying the mass with the acceleration of the hammer. The internal stress was obtained using the output voltage and the sensitivity of PVDF smart sensors. During the tests, the main work completed was the acquisition of the voltage signal from each PVDF sensor via a charge amplifier.

## 3. The Test Results and Discussion

### 3.1. The Time Histories of Impact Force and Stress 

Circular CFSTs with different heights and wall thicknesses were tested by impact tests. The CFST short columns with a thickness of 3 mm were selected to analyze the data of the PVDF piezoelectric sensor and acceleration sensor to obtain a time history curve of the internal stress and drop weight impact force.

**Figure 7:**
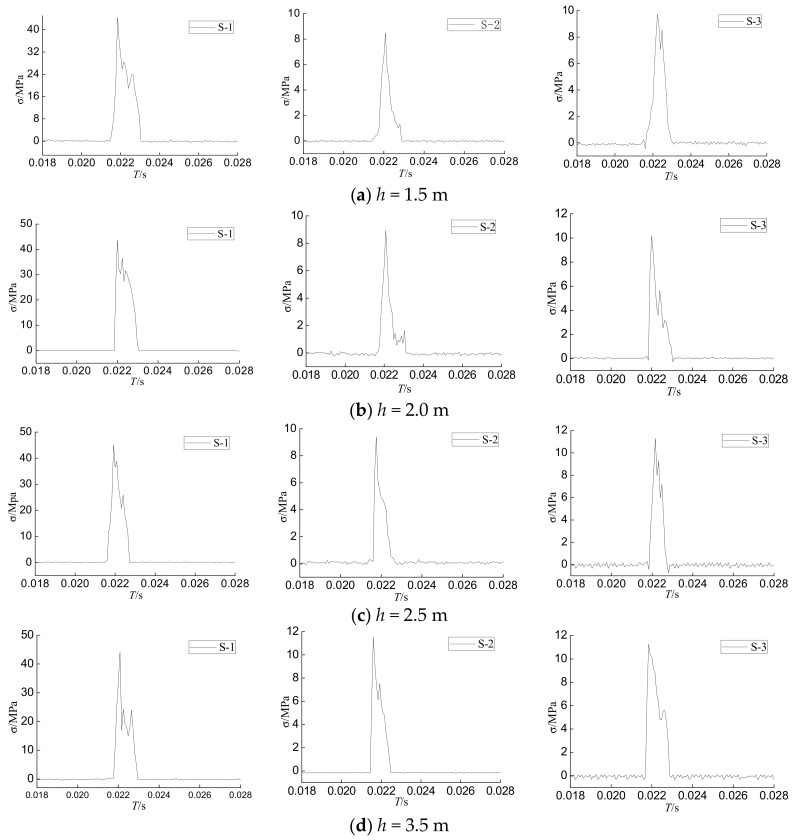
Time history of the internal stresses detected by PVDF sensors.

**Figure 8:**
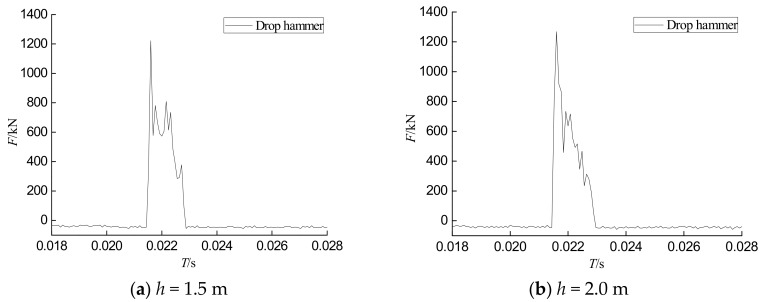

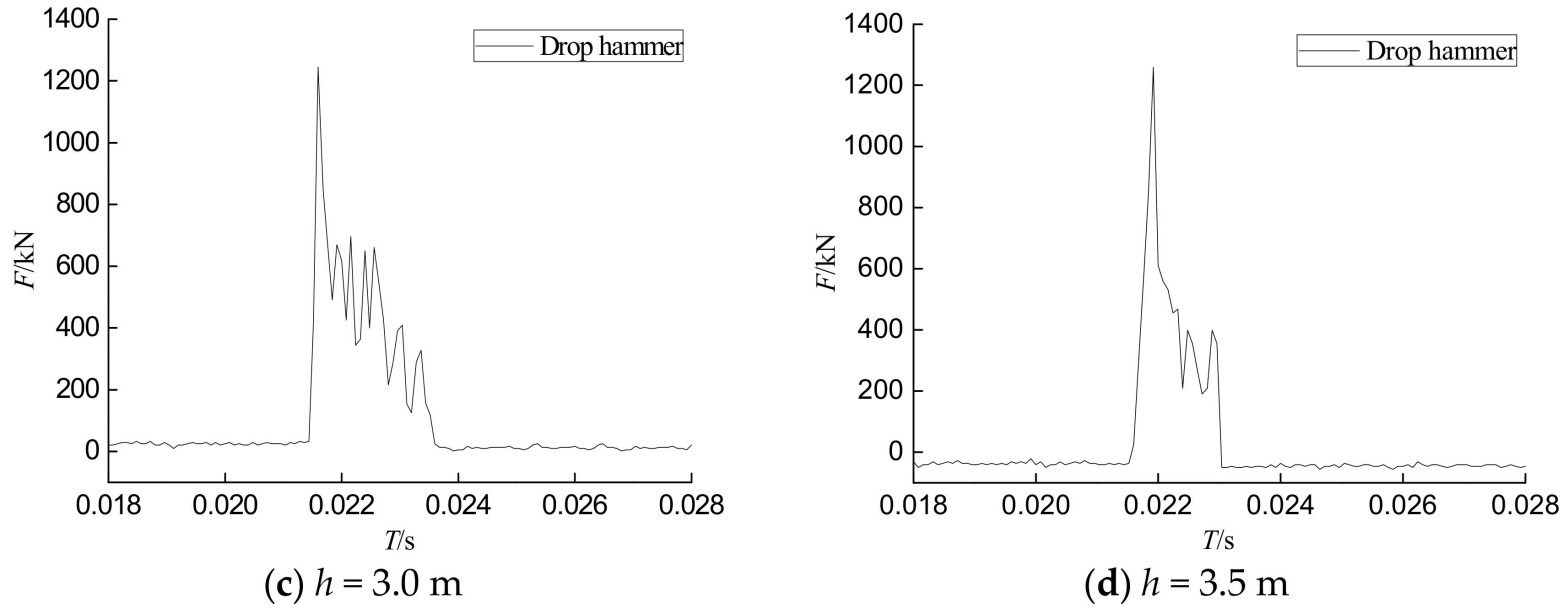
Time histories of the impact forces during different tests.

Under impact loads, different degrees of damage occur inside CFST columns. In addition, the concrete is an anisotropic material. These factors cause the internal forces and the external load to have certain differences under the same impact load. However, it can be seen from Figure 7 and Figure 8 that the overall trend of the internal stress time history curves of the PVDF smart sensor is similar to that of the hammer impact time history curve. They all show three stages, which are the peak stage, the stable stage, and the attenuation stage. In the peak stage, the impact force and internal stress rapidly increase to the maximum level when the hammer is in contact with the specimen. After the full contact between the hammer and the specimen, the impact force and internal stress values decrease and enter the stable stage. With the separation of the hammerhead and the specimen, both the impact force and internal stress rapidly decay to zero, which is known as the attenuation stage.

### 3.2. The Effect of Impact Energy on Impact Stress

**Figure 9:**
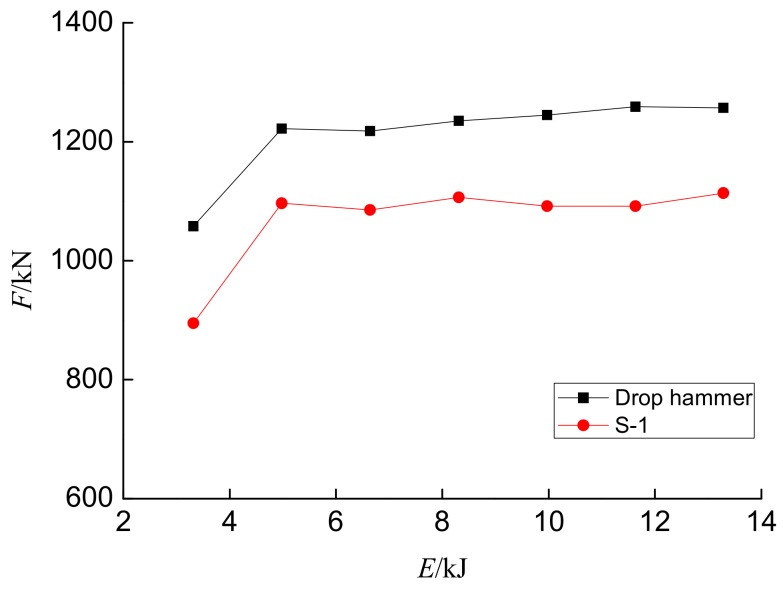
The impact force and internal force detected by sensor S-1 vs. the impact energy.

Figure 9 shows the maximum hammer impact force and the maximum internal force monitored by PVDF sensor S-1 with the impact energy curve. In Figure 9, *F* indicates the maximum impact force under different energy levels, and *E* indicates the impact energy. It can be seen from the figure that maximum internal force of the PVDF smart sensor is basically consistent with that of the hammer impact force. Moreover, the maximum stress of most of the data monitored by response sensors increased with an increase in the maximum impact force. Figure 9 indicates that the use of PVDF smart sensors can effectively reflect the force of structures, and the use of piezoelectric sensors to monitor local stress within structures is feasible.

**Figure 10:**
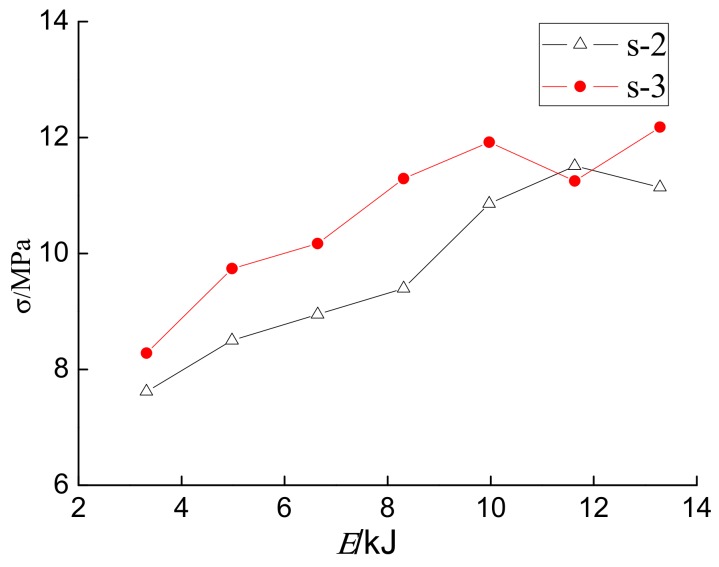
The internal forces detected by sensors S-2 and S-3 vs. the impact energy.

Figure 10 shows the maximum concrete internal stresses monitored by sensors S-2 and S-3 versus the impact energy. In general, it can be seen that both of them have the same trend—an increase in impact energy. When the impact energy is small, the growth rate of the peak values increases rapidly with the increase in energy. However, when the energy reaches a certain value, the peak values of impact force and concrete internal stresses start to saturate.

### 3.3. The Effect of Component Stiffness on Impact Stress

In this paper, three kinds of circular CFST columns with different wall thicknesses were subjected to the impact test. The voltage recorded by the PVDF sensor in each test condition was transformed into the internal stress of the concrete core, as shown in Table 2.

**Figure 11:**
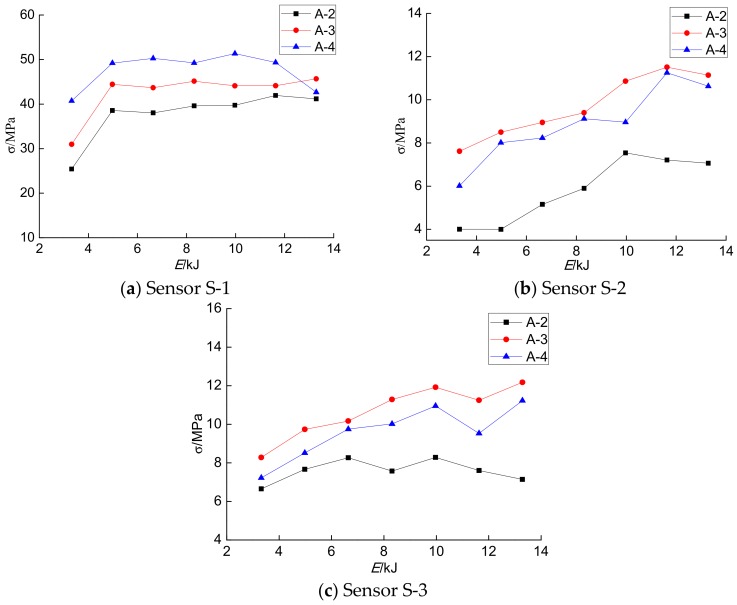
The concrete internal stresses with different wall thicknesses.

By analyzing the concrete internal stress detected by the PVDF sensor in each specimen in Table 2, it can be found from the raw data (except at *h* = 4 m) that the axial stress monitored by the S-1 sensor increases with an increase in the wall thickness of the specimen at the same impact energy, as shown in Figure 11a, which is consistent with fact that the stiffness of the CFST increases with an increase in the wall thickness of the steel tubes. However, the confining stresses of S-2 and S-3 do not increase with the wall thickness of the steel tube. This is because the confining stress is not only related to the thickness of the steel tube wall, but also to the circumferential strain of the steel tube wall. If the strain of the steel tube wall is fixed, the stiffness of the member increases with the increase in wall thickness, and the confining stress of the steel tube wall increases. However, due to the impact load of the members, the circumferential strain of members with larger wall thicknesses is also smaller, which results in the reduction of the confining pressure imposed on the core concrete.

**Figure 12:**
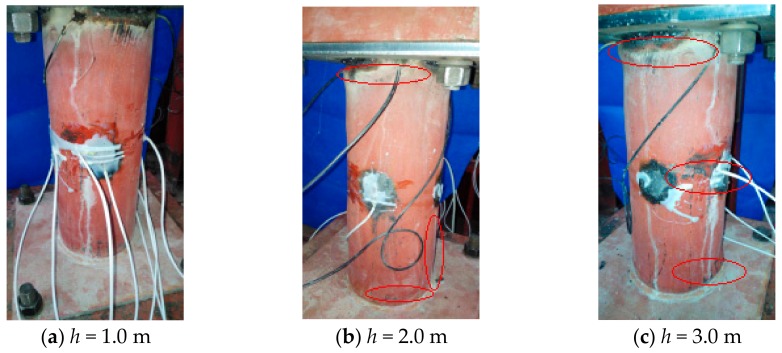
The failure mode of CFST with 3 mm wall thickness.

It was also found that the stresses monitored by the PVDF sensors S-1, S-2, and S-3 no longer increase once the impact energy has reached a certain value. The reason for this is that the internal stress of the specimen increases with the increase of the impact energy when the impact energy is not enough to cause damage to the structure. However, with further increase in impact energy, the local buckling of the specimen leads to a rapid reduction in stiffness, and the internal stress no longer increases with any further increase in impact energy. Local deformation occurs first in the upper part of the specimen and then in the middle and lower parts of the specimen. A wavy, local buckling deformation is formed on the surface of the entire specimen. Eventually, the entire specimen is damaged due to the local buckling or partial cracking, as shown in Figure 12. 

## 4. Finite Element Analysis Model

To simulate the stress conditions of circular concrete-filled steel tubular stub column under impact loads, a nonlinear finite element analysis (FEA) model was established using the ABAQUS/Explicit model including the plastic damage of concrete and the effect of the strain rate.

### 4.1. Model Establishment

The integrated modeling method was adopted in ABAQUS to create the model. In the simulation, the dynamic explicit model with explicit solver was used to simulate the impact force. To utilize resources reasonably, it was considered that the falling hammer and the specimen just contact each other in the initial state without considering the energy loss and the free-falling process of dropping the hammer. The initial speed of the dropped hammer was calculated by the formula, $v\;=\;\sqrt{2gh}$. The mass of the drop hammer in the simulation was 339 kg.

**Figure 13:**
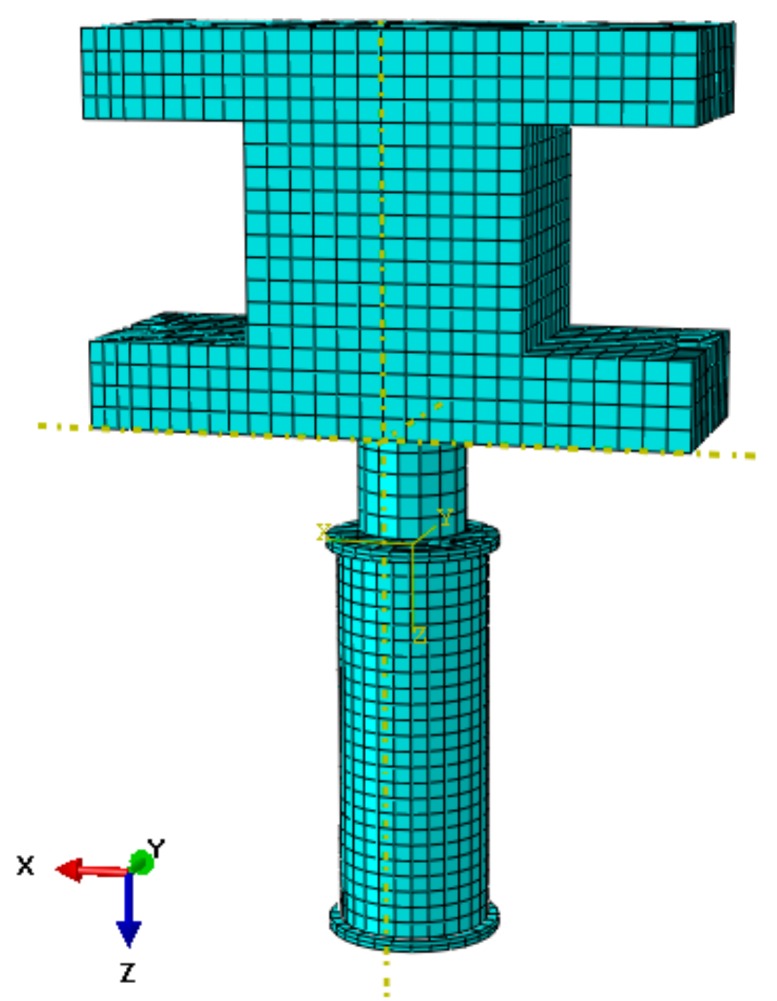
The finite element model.

In the model, the plate, concrete, steel and hammer were simulated by 8-node reduced-integration three-dimensional brick elements (C3D8R). The contact properties of the concrete core with the steel had tangential behavior and normal behavior. Considering the situation of separation after contact between the upper plate and the concrete, the contact properties had only “hard” contact during normal behavior and allowed separation after contact. In view of the slip of concrete and steel tube side walls, the contact properties of these areas had a penalty function. The tie constraint was adopted to simulate the welding connection between the steel tube and the steel plates. With consideration of the computational efficiency, the mesh of the specimen was relatively dense while the hammer was sparse in meshing. The finite element model of the specimen is shown in Figure 13.

### 4.2. Constitutive Models

#### 4.2.1. Constitutive Model of the Steel

In this paper, the elastic-plastic model of steel in ABAQUS was used for the steel material. The stress–strain relationship of steel proposed by Han et al. (2007) has five stages, which includes the elastic stage, the elastic-plastic stage, the plastic stage, the hardening stage and the secondary flow plastic stage.

Under impact loads, the strength of steel will have the following characteristics: the yield strength and ultimate strength of steel increase with an increase in strain rate. At present, there are two kinds of models suitable for computing different strain rates. They are the Cowper–Symonds model and the Johnson–Cook model. However, the load used in this paper is low velocity impact loading, and the Cowper–Symonds model [59] is adopted in this simulation. The dynamic yield stress under different strain-rate is calculated by

$$\sigma_{\mathrm{dy}}\;=\;\sigma_{y}\left[1+{\left(\;\dot{\varepsilon }/D\right)}^{\frac{1}{n}}\right]$$

where $\sigma_{\mathrm{dy}}$ is the dynamic yield strength and $\sigma_{y}$ is the quasi-static yield strength of the steel tube. Meanwhile, it was assumed that the strain rate effect remained unchanged with the strain hardening. D and n are the parameters of strain rate and their values are referenced in the literature: $D\;=\;40.4{\;s}^{-1}$, n = 5. $\;\dot{\varepsilon }$ is the dynamic stress-rate of steel.

#### 4.2.2. Constitutive Relationship of the Concrete Structures

In the actual tests, the stiffness degradation of concrete caused by internal damage is unrecoverable under external loads. In terms of its mechanical properties, the concrete damaged plasticity model in ABAQUS was adopted to simulate the nonlinear behavior of the concrete core under impact loading.

In the selection of the constitutive modeling of the concrete core, the restraint effect of the steel tube on the concrete in the core area was taken into full consideration. The model of concrete compression used in this research was proposed by Liu and Han [60]. This model modifies the peak point of stress and strain and the descending stage in the model proposed by Han [61]. This simplified method is more suitable for the constitutive modeling of core confined concrete under compression.

Under the impact loads, the compressive strength of concrete increased with an increase in the strain rate. The influence of strain rate was considered using the dynamical increasement factor (DIF) in accordance with the European standard (CEB-FIP Model Code 1990) [62].

### 4.3. Comparison of the Finite Element Results with the Experimental Results

**Figure 14:**
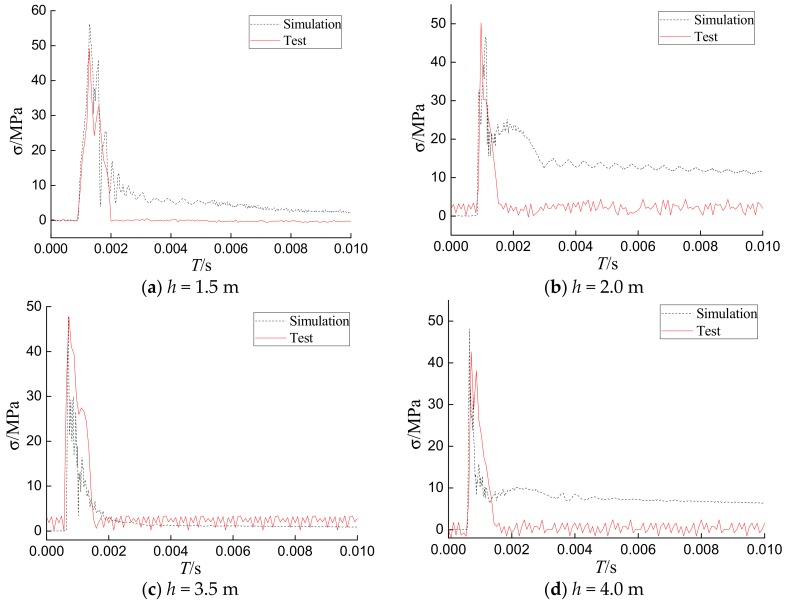
Comparisons of numerical and test results of the internal stress values under different impact heights.

Figure 14 shows the numerical and experimental time–history plots of the stress measured by the S-1 sensor under different impact loads on the concrete filled steel tubular column with 4 mm wall thickness. It can be seen that the stress responses for both numerical and experimental cases were consistent, and the trends were similar. The impact stress obtained by the tests and simulations reached its maximum value in a short period of time. In addition, the maximum level of stress simulated by finite element method had great agreement with the experimental ones. However, the numerical simulation did not fit well after the peak stage. The reason for this is that the internal stress of CFST is very complex, especially under impact loads. When the hammer initially contacts the specimen during an impact, the specimen is a healthy structure without cracks or damage. After the internal stress reaches the maximum in a very short time during impact, cracks and damage start to form. Because of the complexity of the damage inside the specimen and the anisotropy of concrete, the occurrence of random cracks will greatly affect the stress value of the sensor. These factors led to a mismatch between the results of the tests and the finite element simulation after the peak stage. Please note that the general trends of the internal stress and the impact time are similar.

The maximum stress results of both the numerical simulation and experiments at the location of PVDF smart sensors under different drop heights are listed in Table 3. There were minor differences between FEA and experimental results. The error of stress amplitude was within 17% for S-1, within 39% for S-2, and within 6% for S-3. The results show that FEA can be used to predict the evolution of the internal stress of the structure.

## 5. Summary

By embedding the PVDF smart sensor inside the circular CFST column, impact stress monitoring and finite element analysis were carried out, and the following main conclusions were obtained:

(1) For the tested specimens, the impact force–time curve and the stress time history curve were basically the same. The results showed that the embedded PVDF sensor can capture the internal stress of the CFST under an external impact load and effectively monitor the local stress changes in the structure.
(2) It was found that the impact stress increased first and then tended to stabilize with the increase in impact energy by means of impact tests of different CFSTs. The internal concrete stress of a CFST under an impact is not only related to the impact energy, but also to the stiffness of the CFST. When the impact energy is small and the CFST is not damaged, the impact energy is dominant, and the impact stress increases rapidly with an increase in impact energy. With an increase of the number of impacts, the CFST is damaged and the stiffness of the component decreases, and the internal concrete stress tends to stabilize and even decrease under an impact.
(3) By using the finite element simulation of concrete filled steel tubular stub columns under impact load, the numerical prediction of the internal concrete stress and the test results had good consistency. This verifies the feasibility of using a PVDF smart sensor for impact stress monitoring. This monitoring data could be utilized to study the dynamic failure mechanism of concrete-filled steel tubular structures.

In future research, PVDF impact sensors will be embedded in the concrete of the CFST in a distributed fashion to study the internal stress distribution when the structure is subjected to impact loads.

## Figures and Tables

**Table 1:** Sensitivity of each sensor.

Specimens	Sensitivity (MPa/V)		
	S-1	S-2	S-3
A-2	1.288	1.301	1.321
A-3	1.307	1.311	1.285
A-4	1.309	1.312	1.302

Note: The letter A indicates the concrete filled steel tubular column, and the number following A indicates the wall thickness in mm. S indicates the PVDF sensor.

**Table 2:** Maximum stress values of sensors under different impact energy levels.

*h*(m)	*E*(kJ)	A-2 ($\sigma_{max}$/MPa)			A-3 ($\sigma_{max}$/MPa)			A-4 ($\sigma_{max}$/MPa)		
		S-1 (Z)	S-2 (Y)	S-3 (X)	S-1 (Z)	S-2 (Y)	S-3 (X)	S-1 (Z)	S-2 (Y)	S-3 (X)
1.0	3.32	25.41	4.01	6.65	30.97	7.62	8.28	40.72	6.01	7.22
1.5	4.98	38.56	4.00	7.67	44.45	8.5	9.74	49.21	8.02	8.52
2.0	6.64	38.02	5.16	8.27	43.67	8.95	10.17	50.29	8.23	9.75
2.5	8.31	39.60	5.90	7.57	45.15	9.4	11.29	49.23	9.12	10.02
3.0	9.97	39.75	7.54	8.28	44.09	10.86	11.92	51.36	8.96	10.95
3.5	11.63	41.93	7.21	7.60	44.12	11.51	11.25	49.36	11.25	9.53
4.0	13.29	41.17	7.06	7.14	45.67	11.14	12.18	42.67	10.63	11.23

Note: The letter A indicates the concrete filled steel tubular column, the number following A indicates the wall thickness in mm, *h* indicates the impact height, and *E* indicates the impact energy.

**Table 3:** The maximum level of stress of the piezoelectric sensor under different impacts.

*h*/m	σ/MPa					
	S-1 (Z)		S-2 (Y)		S-3 (X)	
	Test	Simulation	Test	Simulation	Test	Simulation
1.0	40.72	48.01	6.01	9.86	7.22	7.65
1.5	49.21	56.26	8.02	11.09	8.52	8.77
2.0	50.29	46.66	8.23	10.83	9.75	9.52
2.5	49.23	45.15	10.12	9.31	10.02	10.50
3.0	51.36	44.06	8.96	9.92	10.95	10.75
3.5	49.36	47.83	11.25	11.37	9.53	12.82
4.0	42.67	48.18	10.63	11.02	14.23	13.90
